# Experience with successful treatment of severe recurrent vulvar adhesions: A case report

**DOI:** 10.3389/fsurg.2023.1052133

**Published:** 2023-02-08

**Authors:** Yingying Bi, Yuhang Chen, Wu Li, Huici Jiang, Jinlong Qin, Jiajing Cheng

**Affiliations:** Department of Gynaecology, Shanghai Fourth People's Hospital, Shanghai, China

**Keywords:** vulvar adhesions, oestrogen, basic fibroblast growth factor, case report, postmenopausal women

## Abstract

Vulvar adhesions are defined as partial or complete adherence of the labia minora and/or labia majora. Vulvar adhesions are rare, especially in postmenopausal women.This article describes a case of postmenopausal recurrent vulvar adhesions successfully treated with surgery. The patient was a 52-year-old woman who had undergone manual separation and surgical adhesion release due to vulvar adhesions, which recurred soon after treatment. The patient then came to our hospital for treatment because of complete dense adhesions to the vulva and laboured urination. The patient received surgical treatment, the anatomical structure of the vulva recovered well, and the symptoms affecting the urinary system disappeared. There was no readhesion during the 3-month follow-up.

## Introduction

Vulvar adhesions have been reported to occur in 0.6 to 1.4 percent of children and are rarer in postmenopausal women, whose incidence is not available (1). Vulvar adhesions may be asymptomatic, and some patients seek medical attention due to urinary tract symptoms or vulvar discomfort. Urinary tract symptoms include dysuria, urgency, urinary retention, and recurrent urinary tract infections. Symptoms of vulvar discomfort include genital itching, vulvar pain, and dyspareunia. Vulvar adhesions can also prevent doctors from performing a comprehensive gynaecological examination, masking symptoms such as vaginal bleeding and palpable lumps. Though vulvar adhesions have a serious impact on women's physiology and psychology, because the site of the disease is private, patients often refuse to seek medical treatment. Also, due to the rarity of vulvar adhesions in postmenopausal women, there are currently no practical guidelines or standard treatment strategies to guide the treatment, there is a lack of effective means for the high recurrence rate after treatment, and clinicians lack experience treating such patients. We share our experience in this treatment case to provide a reference for the clinical treatment and prevention of recurrence of vulvar adhesions in postmenopausal women.

## Case report

Medical history: The patient was a 52-year-old female with regular menstrual cycles, moderate menstrual volume, and no history of dysmenorrhea. She has been in natural menopause for 5 years and has denied abnormal vaginal bleeding after menopause. In 2017, the patient underwent separation surgery in a foreign hospital because of partial adhesion of the labia minora. The surgery was carried out under local anesthesia, through manual separation of the adhesions. The vulva, vagina and urethra opening were completely exposed after the surgery,but the vulva readhered 2 weeks after treatment. In 2019, the patient returned to another hospital due to vulvar adhesions and underwent surgical labia minora resection and vulvar adhesion release.The vulva, vagina and urethra opening were completely exposed after the surgery. During this period, the patient's lung nodule biopsy report suggested lung cancer *in situ*, and she was required to be discharged for thoracoscopic surgery. Medicine, less than 1 month after the operation again vulvar adhesions. This time, the patient came to our outpatient clinic for treatment due to dense adhesion of the vulva and laborious urination.

**Figure 1:**
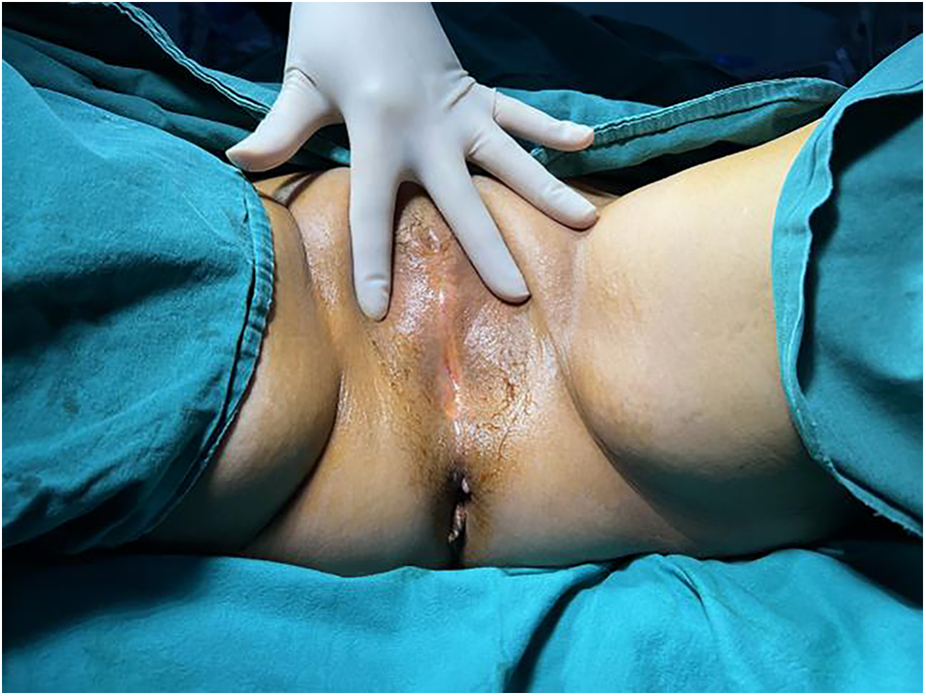
Dense adhesion of the labia from the clitoris and to the perineum.

Gynaecological examination revealed vulvar atrophy, dense adhesion of the labia from the clitoris and to the perineum, adhesions close to the urethral opening and vaginal opening, and only a pinhole-sized opening (Figure 1).

Preoperative preparation: beginning 1 week before surgery, the patient was instructed to apply oestrogen cream to the perineum before going to bed every day.

**Figure 2:**
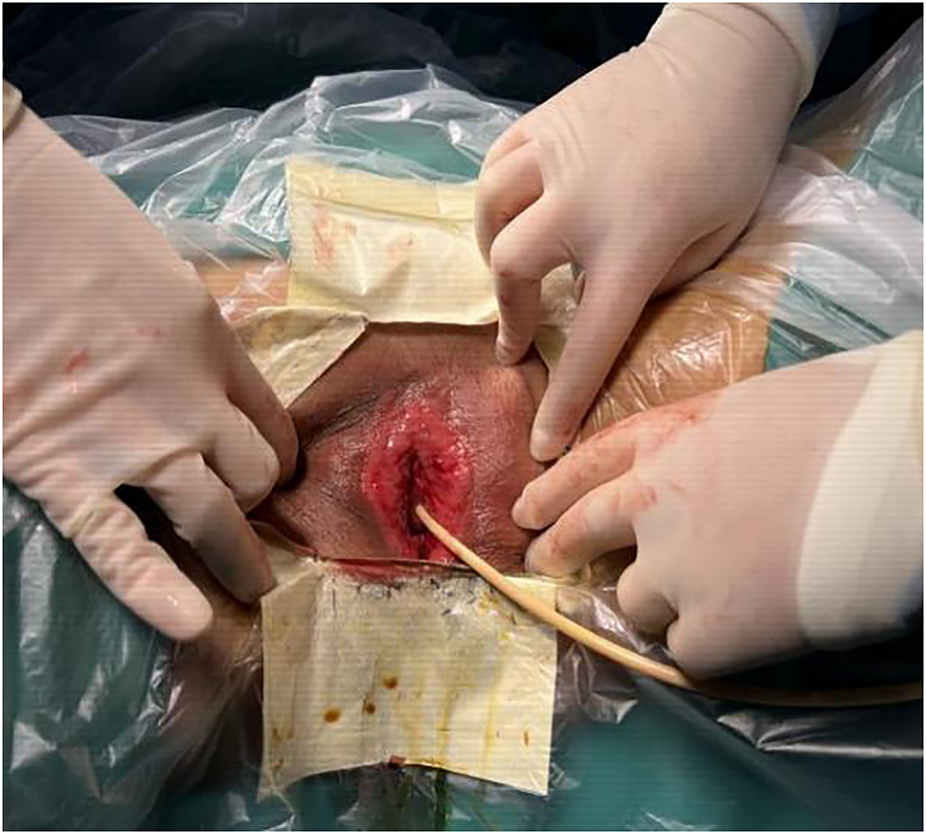
The skin of the adhesion separation wound was thin, with little blood oozing.

**Figure 3:**
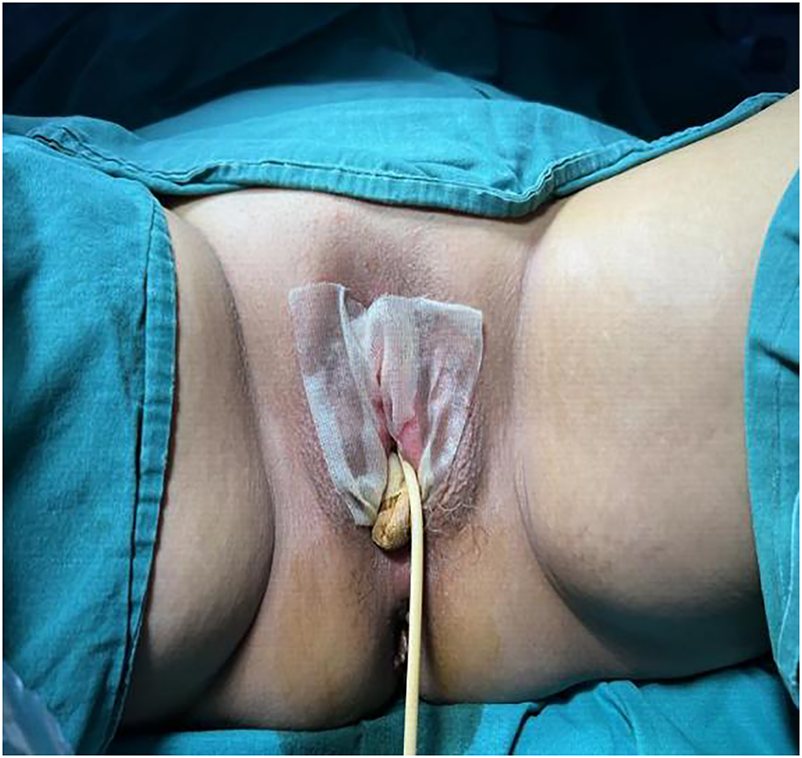
A piece of vaseline gauze was used to cover the vulvar adhesion and separation surface.

Surgical procedure: The patient was taken to the bladder stone surgical site, and intravenous anaesthesia was applied. The adhesions were bluntly separated with vascular forceps at the opening, and the dense adhesions were separated with an ultrasonic knife. The vaginal opening, urethral opening, and clitoris were exposed in turn to check for vaginal patency. The skin of the adhesion separation wound was thin, with little blood oozing (Figure 2). The operation went smoothly, and the intraoperative blood loss was 2 ml. A piece of Vaseline gauze was placed in the vagina for indwelling catheterization, and a piece of Vaseline gauze was used to cover the vulvar adhesion and separation surface (Figure 3).

**Figure 4:**
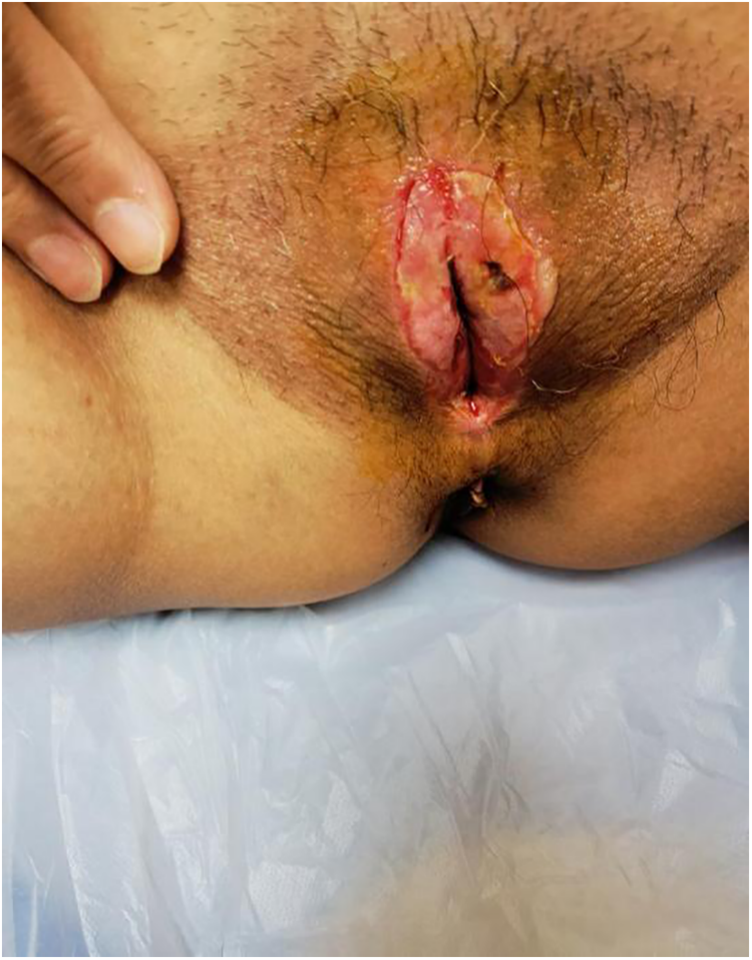
Healthy granulation tissue with a small amount of exudate.

Postoperative dressing change: During the first week post-surgery, the wound surface was disinfected with iodine every day and oestrogen cream and levofloxacin antibacterial ointment was applied to the wound surface The urinary catheter was removed 2 days after surgery. The patient experienced discomfort including frequent urination and incontinence. Healthy granulation tissue with a small amount of exudate was seen 1 week after surgery (Figure 4).

**Figure 5:**
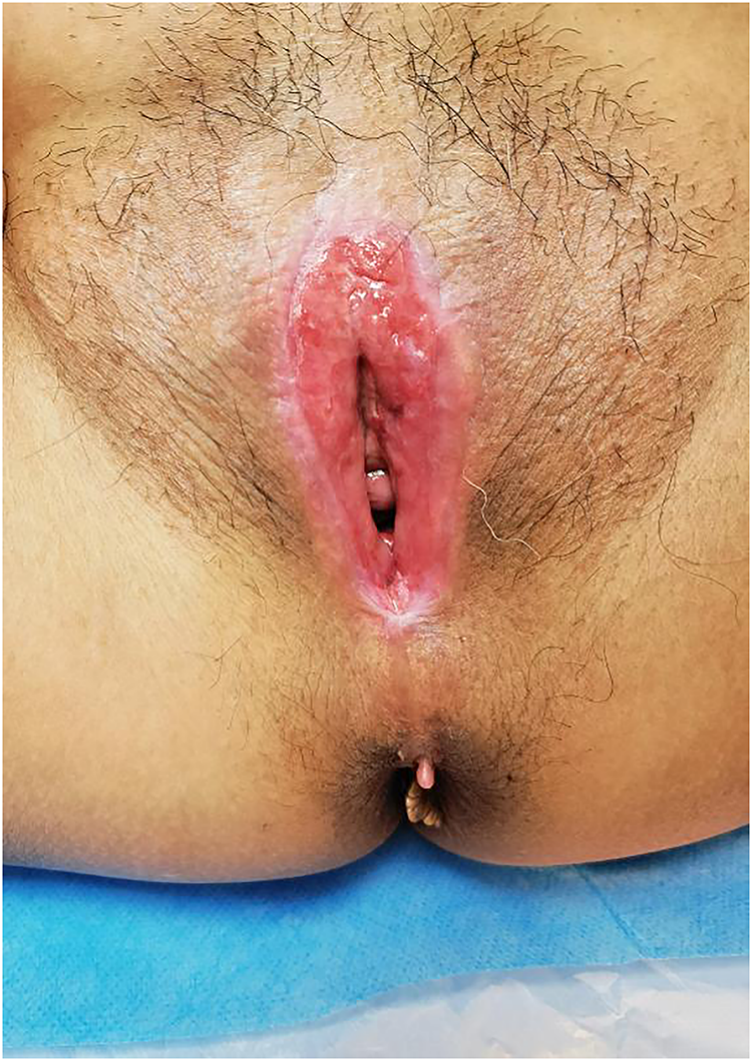
The separated wound surface was healed and formed a smooth surface.

**Figure 6:**
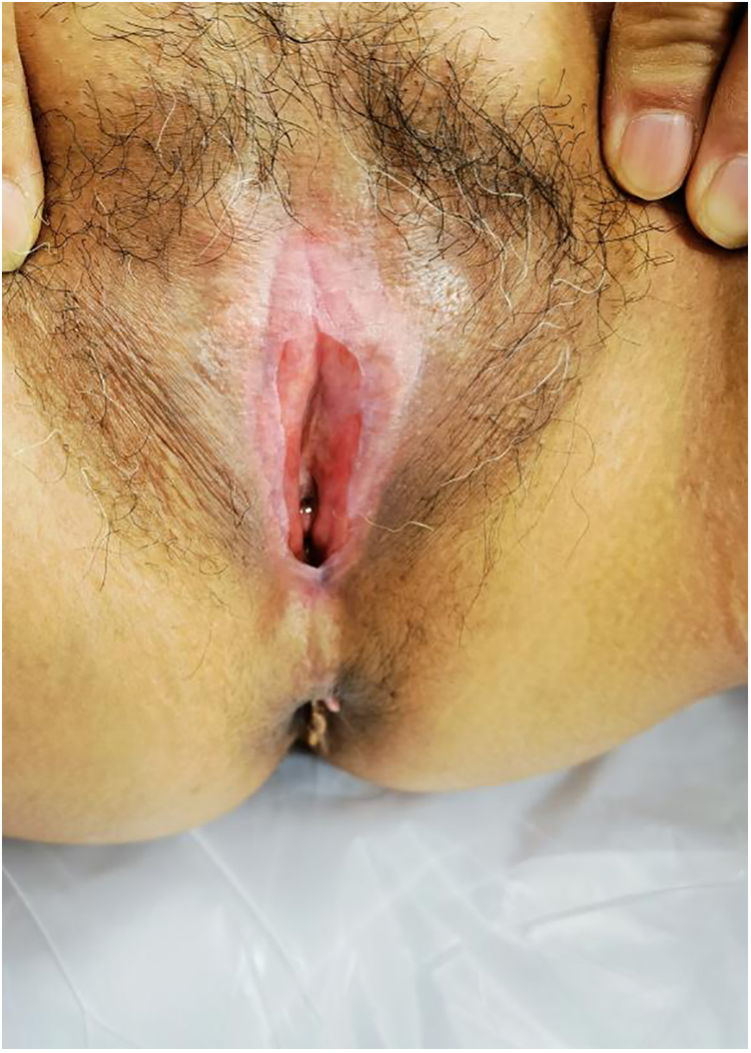
Three weeks after the operation the wound was basically healed.

After the first week post-surgery, the recombinant bovine basic fibroblast growth factor solution was directly sprayed on the wound surface. The separated wound surface was healed and formed a smooth surface 2 weeks post-surgery (Figure 5). Three weeks after the operation, the wound was basically healed (Figure 6).

Follow-up: No surgical complications occurred. The patient was followed up with at the outpatient clinic at 1 month and 3 months after the operation. The anatomical structure of the vulva recovered well, and no vulvar adhesions recurred. Self-reported urinary symptoms disappeared completely.

## Discussion

Vulvar adhesions, also known as labial adhesions, refer to partial or complete adhesions of the labia minora or majora, mostly located near the clitoris. The reported incidence of vulvar adhesions in children is 0.6–1.4%, with a peak incidence between 13 and 23 months. Vulvar adhesions are a rare disease in postmenopausal women, and their incidence in postmenopausal women has not been reported in significant numbers (1, 2).

The aetiology of vulvar adhesions is unclear, but low oestrogen levels and lack of sexual activity are possible causes. Atrophic changes in the mucous membranes and skin of the vulva occur due to low levels of physiological oestrogen. Long-term inflammation can lead to the exfoliation of epithelial cells on the vulvar surface, making the exfoliated surface of the labia adhere and fuse during the healing process (3). Atrophic changes, along with chronic inflammatory changes, lead to vulvar adhesions, with partial or complete obstruction of the urethra and/or vagina, as the condition progresses. Aggravating factors include chronic inflammation due to poor hygiene, eczema, seborrheic dermatitis, lichen planus or sclerosis, local trauma, and recurrent urinary tract infections (4–6). In this case, the patient's age at menopause was 47, earlier than the average age of menopause (7). After menopause, she had not received hormone replacement therapy and was in a long-term low oestrogen state. In addition, due to family reasons, the patient and her husband were not having intercourse for 4 years. We believe that these reasons caused the patient's vulvar adhesions. In addition, during the postoperative follow-up, we found the phenomenon of hypopigmentation of the patient's vulva, and this pathological change of leukoplakia vulvae may be the basis of the adverse outcome of vulva adhesion in these patients. Therefore, early diagnosis of this pathological change is very important. For this patient, we negotiated a follow-up vulva biopsy to clarify the pathological changes.

Vulvar adhesions are clinically diagnosed and do not require laboratory or imaging studies. Vulvar adhesions can range from affecting only part or all of the labia minora to covering the urethral opening and vaginal opening. Doctors can find abnormalities of female external genitalia through gynaecological examination, which is the simplest and most important diagnostic tool for vulvar adhesions. The severity of vulvar adhesions varies with the remaining length of the vaginal opening. In this case, we examined the patient's external genitalia. The patient's vulva was atrophied, and the labia were densely adhered from the clitoris to the perineum. The adhesions closed the urethral opening and vaginal opening, and only a pinhole-sized opening was seen. According to the gynaecological examination results, the patient's vulvar adhesions were diagnosed.

Vulvar adhesions may be asymptomatic, and some patients may have symptoms of urinary tract or vulvar discomfort. Urinary tract symptoms include dysuria, urgency, urinary retention, and recurrent urinary tract infections (8, 9). Vulvar adhesions are rare in postmenopausal women, and voiding dysfunction due to vulvar adhesions is even rarer and has been reported in only a few case studies (10, 11). Vulvar discomfort includes genital itching, vulvar pain, dyspareunia, etc. (9, 12). If there are no symptoms of vulvar adhesions, some scholars suggest follow-up observation, and some scholars suggest early intervention to avoid clinical symptoms caused by vulvar adhesions (13–15). In prepubertal cases of vulvar adhesions, usually only the adhesions of the labia minora are found. In postmenopausal cases, adhesions of the labia majora often occur as well. Vulvar adhesions prevent doctors from conducting a comprehensive gynaecological examination, mask vaginal bleeding symptoms and palpable masses and thus may hinder the diagnosis of gynaecological malignancies such as cervical cancer and endometrial cancer (16). In the case we reported, the patient presented with severe vulvar adhesions that resulted in laborious urination, and examination revealed that the adhesions completely covered the urethral opening and vaginal opening. During the annual physical examination, the patient was unable to undergo transvaginal uterine appendage ultrasound examination, cervical exfoliative cell examination and human papillomavirus examination. Therefore, we suggest that vulvar adhesions in postmenopausal women should be treated with active early intervention to restore normal anatomy and prevent recurrence.

Depending on the degree of vulvar adhesions, treatment includes topical oestrogen creams, manual separation of the adhesions, or different modalities of surgical release of the adhesions. Treatment of mild cases includes topical oestrogen cream, which is safe and effective for the treatment of vulvar adhesions (14). Topical steroids are indicated for inflammatory conditions such as vulvar lichen planus or sclerosis. A case study demonstrated that persistent vulvar adhesions can be treated with simple episiotomy as long as the inflammation is controlled (17). Topical therapy is not always successful, and surgery for adhesion release is required after conservative treatment fails. A period of topical treatment is recommended prior to surgical treatment. In this case, the patient was instructed to apply oestrogen cream preoperatively, which increased the local skin elasticity, and there was almost no bleeding during the operation.

Although various treatment options for vulvar adhesions have been proposed, there are currently no clearly recommended measures to reduce recurrence. Recurrence of vulvar adhesions is common regardless of the method of treatment used. A study of 71 girls with vulvar adhesions by Ellen Wejde et al. (6) showed that both topical oestrogen therapy and manual separation of adhesions had high long-term recurrence rates. Adhesion recurrence occurs in 14 to 20% of patients following manual dissection or surgical release (5). For postmenopausal intractable vulvar adhesion cases, preventing postoperative recurrence is also a difficult problem. The use of oestrogen creams and regular manual separation of the vulva are important, especially for those who are not sexually active. Johnson et al. (18) treated an elderly woman with refractory vulvar adhesions. After vulvar adhesion release surgery, a full-thickness flap was isolated from the patient's thigh for grafting, which successfully prevented vulvar contraction and scarring. Postoperatively, the patient did not develop adhesions again. However, this treatment method is expensive and technically demanding. There are few reports using this method for the treatment of postmenopausal vulvar adhesions.

Fibroblast growth factors are a class of secreted polypeptide ligands that are widely used in the treatment of burns, chronic wounds (including chronic ulcers), and fresh wounds (traumatic and surgical wounds). Animal experiments show that this product can promote capillary regeneration, improve local blood circulation, and accelerate wound healing. An animal experiment demonstrated that recombinant bovine basic fibroblast growth factor could promote wound healing after thorough debridement in New Zealand rabbits (19). Hong-Xia Du et al. investigated the effect of recombinant bovine basic fibroblast growth factor treatment on scar relief and inflammatory cytokines in patients with atrophic acne scars. Recombinant bovine basic fibroblast growth factor as an adjuvant therapy for CO2 fractional laser treatment can effectively relieve atrophic acne scars and local inflammatory reactions, with good curative effects and few adverse reactions (20). Recombinant bovine basic fibroblast growth factor for the treatment of burns, granulation tissue formation and epidermal regeneration can effectively shorten the wound healing time, improve the quality of wound healing, and reduce the hospitalization time, cost and emotional burden of patients.

At present, there is no report in the literature on the application of recombinant bovine basic fibroblast growth factor in wounds after vulvar adhesion surgery. In our case, the patient started using the recombinant bovine basic fibroblast growth factor solution when he saw healthy granulation tissue with a little exudate on the wound 1 week after surgery, and by 2 weeks after the operation, the isolated wound healed, a smooth surface formed, and the wound healing was satisfactory. The patient was followed up with for 3 months, and no readhesion occurred.

## Conclusions

The incidence of vulvar adhesions in postmenopausal women is low, and clinicians lack experience and coping strategies in treating such patients, especially in preventing postoperative recurrence. The patient reported in this case experienced two recurrences of vulvar adhesions. The wounds healed quickly after our treatment of the second occurrence, and the effect was satisfactory. The adhesions did not recur during follow-up. This experience suggests we can draw conclusions regarding treatment of this type of disease. For postmenopausal women with vulvar adhesions, it is recommended to release the adhesions by surgery. Local use of oestrogen before surgery can greatly reduce intraoperative bleeding and the use of oestrogen and antibiotic creams can be applied to the wound surface post-surgery. After the granulation tissue is formed, recombinant bovine basic fibroblast growth factor solution can be directly sprayed on the wound surface to promote the rapid healing of the wound surface and form a smooth surface. This treatment reduces adhesion recurrence.

## Data Availability

The original contributions presented in the study are included in the article/Supplementary Material, further inquiries can be directed to the corresponding authors.
